# The Challenges of Working with Legacy NMRs

**DOI:** 10.1021/acscentsci.4c00160

**Published:** 2024-02-14

**Authors:** Celia Arnaud

In 2014, Agilent Technologies announced that it would stop selling nuclear magnetic resonance systems. Agilent, which had acquired its NMR rival Varian in 2010, continued to fulfill existing orders, so the last of its systems wasn’t installed until 2016. The owners of NMR spectrometers manufactured by either firm now face an uncertain future.

Agilent says it has no plans to stop supporting the instruments, but customers know that they’re living on borrowed time: when the company decides to leave the field completely, they will face the choice of maintaining the systems themselves, finding third-party companies to provide service, or upgrading their systems with newer control consoles.

NMR systems are core tools for research chemists. In proton NMR spectroscopy, samples are placed in a spinning probe in a strong magnetic field—typically between 400 and 900 MHz. The nuclei of hydrogen atoms in the sample align with the magnetic field. To detect the atoms, the sample is subjected to electromagnetic pulses. The pulses perturb the alignment, an action that causes emission of electromagnetic waves. These carry information about the chemical environment of each of the hydrogen atoms, which makes it possible to distinguish one bonding arrangement from another and deduce the structure of the sample molecules.

To function properly, the magnet typically must be cooled with liquid helium. Researchers use a console to control the pulses and detect the nuclear responses.

So far, Agilent’s NMR service is going strong. David Rice has been director of the NMR facility at the University of California, Merced, since 2016. He was previously an application scientist at Varian and Agilent. “The UC Merced NMR facility has had a service contract with Agilent for my Varian and Agilent instruments since I joined here,” he says. “They’ve given me excellent advice and come through with parts for me when I needed them.”

The High-Resolution NMR Facility at Washington University in St. Louis (WUSTL) is home to one of the last NMR systems Agilent sold. In addition to that 600 MHz instrument, the facility runs four other Varian legacy instruments, according to Manmilan Singh, director of the facility.

**Figure:**
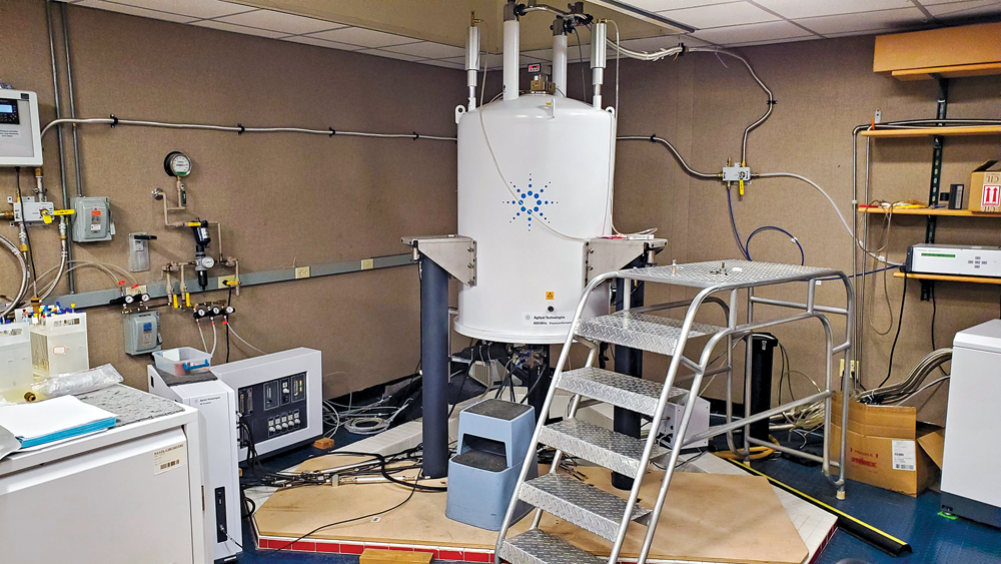
This NMR at Washington University in St. Louis was one of the last sold by Agilent Technologies. Credit: Manmilan Singh/Washington University in St. Louis

Singh still calls Agilent when he needs a part to repair a machine. “The newer instruments, if something goes wrong with them, they definitely have the parts for it,” he says. “Some of the older ones, they can find the part for you, but it takes a while sometimes. Then you have to start exploring other avenues.”

One avenue is do-it-yourself maintenance. The High-Resolution NMR Facility has multiple defunct Varian systems that researchers mine for parts. Singh is “extremely good with hardware,” says Sophia Hayes, Vice-Dean of Graduate Education and an NMR researcher in WUSTL’s chemistry department. “His skills with NMR hardware are extraordinary. Most people have one console, and when something breaks, there’s no backup.”

Daniel Holmes, who runs the NMR facility at Michigan State University, takes a similar DIY approach. “Mostly I rely on myself and my years of experience with these systems. I’ve been doing this for 20-some years, so I know what tends to break,” he says.

At both WUSTL and Michigan State, the NMR facilities stockpile old consoles after upgrades to scavenge them for parts. And they don’t keep just their own systems. Some universities give away old consoles when they upgrade because they don’t have the storage space. “We will snap them up, so I have a lot of spare parts,” Holmes says.

**Figure:**
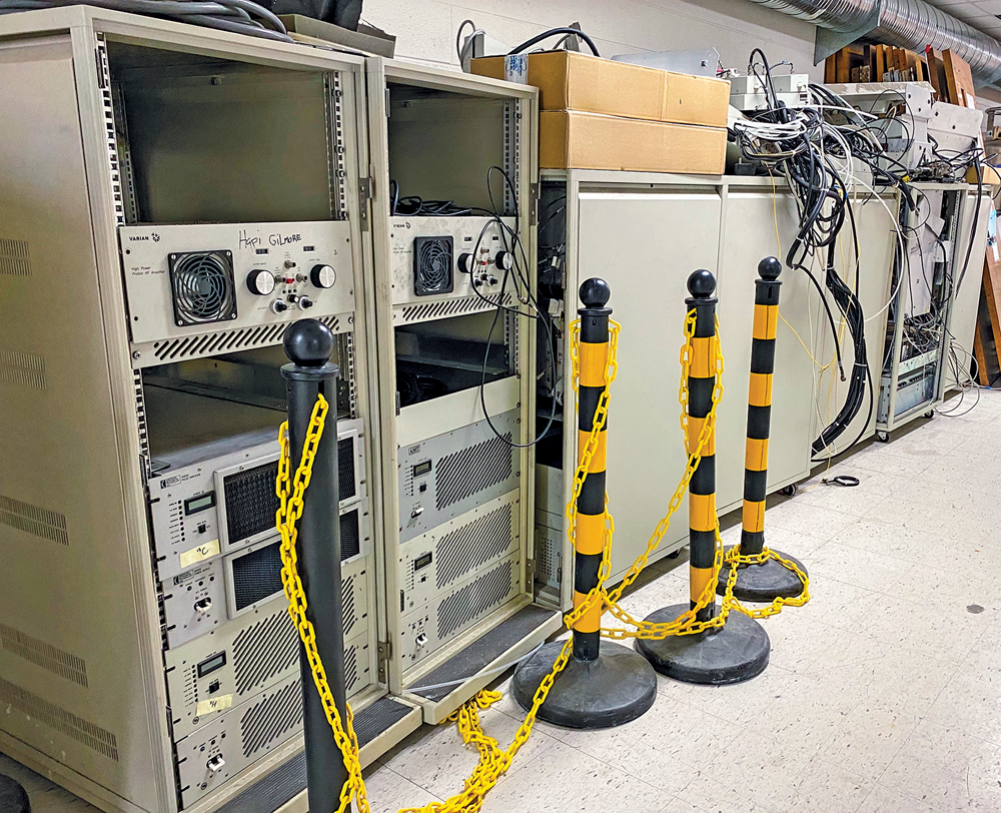
Old NMR consoles, such as these stored at Michigan State University, can be scavenged for parts. Credit: Daniel Holmes/Michigan State University.

For people looking to keep their existing systems running, user groups can provide advice. After Agilent announced that it was leaving the NMR market, Rice helped organize IVAN—which originally stood for International Varian–Agilent NMR—as a place for Varian and Agilent users to ask and answer questions.

IVAN has since expanded into a general NMR discussion forum; the acronym now stands for Inspiring a Versatile and Agile NMR Community. The group sponsors user meetings immediately before the annual Experimental NMR Conference.

Properly maintained magnets can last for decades. But as control technology improves, an older console can become a hindrance. Users with legacy NMR spectrometers have the option of installing either new or refurbished consoles.

“In a 15-year-old NMR spectrometer, the magnet still has many, many years of life to it,” says Jon Webb, the founder and CEO of MR Resources, a company that sells late-model, reconditioned spectrometers, consoles, and magnets. Webb is also one of the founders of IVAN. A customer with such a spectrometer “might choose the path of a reconditioned console,” he says. “We would sell them a console new to them, which would be 2 or 3 or 4 years old and would have a significantly lower price point than a brand-new console.”

Or people can opt for a brand-new console. JEOL, Bruker, and Q One Tech are the remaining suppliers of large NMR instruments. Earlier this year, JEOL launched a reconsoling initiative for legacy NMRs. “Some of those NMR systems are pushing 20 years old, so the electronics in the computers are getting dated, and failure modes are increasing,” says Michael Frey, an emeritus NMR product manager at JEOL who was involved in developing the initiative.

“It’s very cost effective to just pull out the console, leave the magnet, and put a new console on it,” Frey says. “The large investment is the magnet. The console is, depending on the field, anywhere from 50% down to 20% of the value of the system. So that’s a big cost savings.” Despite being less expensive than an entirely new system, a new console is still in the six-figure range and can be out of reach for smaller institutions.

For reconsoling, the system is stripped of everything but the magnet. That means removing the old console, shim system, and probes and installing new components. The shim system controls the homogeneity of the magnetic field, and the probes hold samples and contain the electronics used for exciting nuclei and detecting NMR signals.

“It’s like a standard NMR system installation after that, although you’re not going through the hassle of having to go through a complete magnet installation,” Frey says. “It’s usually much quicker, typically less than a week, and you’re back up and going with a brand-new system with typically better performance than what you had before, as well as greatly increased reliability.”

Not every magnet is a good candidate for reconsoling. “We ask the customers to fill out some documentation because we have to know what the magnet is. They are mechanically different,” Frey says. “There are a few very strange magnets that only a couple of versions of were sold.” In addition, if a magnet has been quenched or shut down, the likelihood is slim of being able to make it work with a new console.

While researchers deal with the practical ramifications of Agilent’s exit, WUSTL’s Hayes is considering the broader implications. “I think we might see changes, because what this has shown is that it’s like a single-point-of-failure model. We are now in a situation where hardware with very high capital equipment costs is purchased, only to learn that the company may choose not to be in this business within a year or two thereafter,” she says.

NMR instruments are unique in their longevity, Hayes notes. “In many cases we have been fortunate as a department to keep them for 20 or 30 years. So what do you do in terms of robust decision-making when the landscape for vendors of such equipment is so uncertain?”

Hayes predicts that in the next decade or two there could be a shift toward benchtop instruments for routine analysis in synthesis laboratories—both to avoid the large purchases and to circumvent difficulties with the helium market. But until then, she adds, “every research-oriented chemistry department needs an NMR—at least one, if not two.”

## Celia Arnaud is a freelance contributor to

Chemical & Engineering News, *the independent news outlet of the American Chemical Society. A version of this story appeared in C&EN*.

